# How to Make Nerve Bristles

**Published:** 1878-11

**Authors:** Theodore F. Chupein


### ARTICLE VII.

### *How to Make Nerve-Bristles.*

BY THEODORE F. CHUPEIN, D. D. S.

Take an old excavator; remove the temper by heating it red-hot; file this down with an ordinary bench file as small as possible. When the file becomes too coarse to reduce it further, hold the handle of the instrument between the fingers so that the working point or end will lay flat on any level surface, say a piece of soft pine wood; continue the attenuation of the working end by rubbing coarse emery-paper first, and fine emery-paper secondly over this, turning the handle of the instrument round and round with the fingers of the left hand, in which it is held until the fineness of a hair is attained; lastly, with a slip of Arkansas oil-stone, continue the rubbing until all the scratches of the fine emery-paper are removed. Next, to make the hook. It would be useless to attempt to form this with the ordinary, or even with an exceedingly small pair of flat-nose pliers, as they never could form a hook sufficiently acute at the turning point. For this purpose take the handle of an old separating file, and with a bench file bevel off the end in the shape of a carpenter's chisel. Now lay the excavator, which has been prepared as directed, on this, allowing only as much of the hair-like end to extend beyond as you wish to bend over to form the hook. Hold it firmly on to the piece of separating file, prepared as directed, close up to the beveled part, with a pair of pin-vices, and with a knife-blade or any fine instrument the projecting point may be turned over so as to form a sharp angular hook. Now lay this on a small bench-anvil, and with a minute rivetting-hammer and a strong magnifying glass the hook may be flattened from the round form in which the emery-paper left it. By means of the magnifying glass and the Arkansas oil-stone slip, any little roundness may be reduced so as to form the attenuated point into a sharp angular hook. To temper, lay a long drop of water on a piece of metal, holding this in the left hand very near the blaze of the spirit-lamp. Now hold the instrument in the blaze, and as soon as it is red-hot, (which, from its extreme attenuation, occurs almost as soon as it enters the blaze,) shove it into the water near by. To reduce to a blue or spring temper, heat a piece of metal and lay the hardened bristle on it; watch the color, and plunge it into the water, held near at hand, as soon as possible. The tempering is the most delicate part of the process.

An old barbed nerve-broach may be worked in the same way, and, when finished, mounted in a handle.—*Dental Office and Laboratory.*

